# All-Aqueous Bicontinuous Structured Liquid Crystal Emulsion through Intraphase Trapping of Cellulose Nanoparticles

**DOI:** 10.1021/acs.biomac.2c01177

**Published:** 2022-12-08

**Authors:** Shasha Guo, Han Tao, Guang Gao, Sameer Mhatre, Yi Lu, Ayako Takagi, Jun Li, Lihuan Mo, Orlando J. Rojas, Guang Chu

**Affiliations:** † School of Chemistry and Chemical Engineering, State Key Laboratory of Pulp and Paper Engineering, South China University of Technology, Guangzhou 510640, China; ‡ Bioproducts Institute, Department of Chemical & Biological Engineering, Department of Chemistry and Department of Wood Science, The University of British Columbia, Vancouver, British Columbia V6T 1Z3, Canada; § Bio-based Colloids and Materials, Department of Bioproducts and Biosystems, School of Chemical Engineering, Aalto University, Vuorimiehentie 1, Espoo 02510, Finland; ∥ Department of Cellular and Physiological Sciences, Life Sciences Institute, University of British Columbia, Vancouver, British Columbia V6T 1Z3, Canada

## Abstract

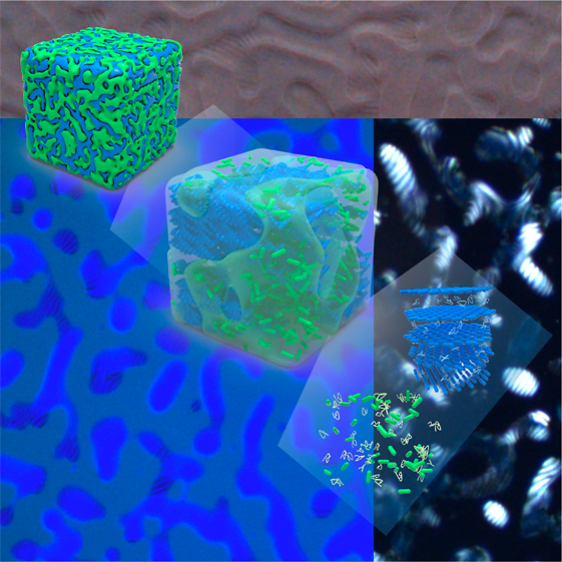

Here, we describe the all-aqueous bicontinuous emulsions with cholesteric liquid crystal domains through hierarchical colloidal self-assembly of nanoparticles. This is achieved by homogenization of a rod-like cellulose nanocrystal (CNC) with two immiscible, phase separating polyethylene glycol (PEG) and dextran polymer solutions. The dispersed CNCs exhibit unequal affinity for the binary polymer mixtures that depends on the balance of osmotic and chemical potential between the two phases. Once at the critical concentration, CNC particles are constrained within one component of the polymer phases and self-assemble into a cholesteric organization. The obtained liquid crystal emulsion demonstrates a confined three-dimensional percolating bicontinuous network with cholesteric self-assembly of CNC within the PEG phase; meanwhile, the nanoparticles in the dextran phase remain isotropic instead. Our results provide an alternative way to arrest bicontinuous structures through intraphase trapping and assembling of nanoparticles, which is a viable and flexible route to extend for a wide range of colloidal systems.

## 1 Introduction

Colloidal self-assembly is ubiquitous in nature which provides an important means to build materials with hierarchical structures. To achieve this, emulsions are particularly valuable for their ability to organize particles and molecules at the fluid–fluid interface, creating a vital bond of colloidal self-assembly between nano- and microscales.^1^ Emulsions are multiphase systems with one liquid dispersed into the other, displaying many applications ranging from common household products to fancy optical display materials.^2−4^ Compared to classic emulsions with spherical droplets, bicontinuous emulsion displays two tortuous interpenetrating fluid domains and a three-dimensional (3D) liquid network,^5^ in which both molecules (e.g., surfactants, lipid, and block copolymers, etc.) and nanoparticles can be used to stabilize the bicontinuous morphology.^6−10^ Examples of such bicontinuous structured emulsions include bicontinuous interfacially jammed emulsion gels (bijels),^11^ bicontinuous intraphase jammed emulsion gels (bipjels),^12,13^ and polymer blends.^14^ In these systems, the two immiscible components along with dispersed colloidal particles can form tortuous liquid channels by arresting the solvent spinodal decomposition process, while the particles are kinetically trapped either within one liquid domain or at the interfacial region, creating colloidal self-assembly of nanoparticles at varying range scales. Research into such bicontinuous structured emulsion is not only interesting for revealing the non-equilibrium phase separation process but also attracts a broad interest for their various kinds of applications.^15−18^ Up to now, extensive works have been focused on the fluid–particle and particle–particle interactions to control the tunability, scalability, versatility, and stability of bicontinuous emulsions;^19^ however, the hierarchical colloidal self-assembly of nanoparticles within percolating bicontinuous structures has so far remained poorly understood.

Liquid crystals (LCs) have preserved long-range orientational order of crystal and flowing mobility of liquid, acting as an essential role in material design and modern technology.^20^ Confining anisotropic LC fluid into an emulsion system is widely explored as a LC emulsion and displays distinctive nano- and microstructures.^21−23^ When the prepared LC emulsion shows microscale droplet morphology, the LC orientational order can be frustrated in confined spherical geometries by the interplay between boundary conditions and energy minimization, yielding complex behaviors to control microscale colloidal self-assembly with super structures that are distinguished from the bulk phase.^24−28^ Interestingly, some colloidal particles can self-align along a common axis at high concentrations, resulting in the anisotropic LC phase with long-range ordered particle assembly. For example, cellulose nanocrystals (CNCs) are charged, rod-like nanoparticles that can self-assemble into a cholesteric LC phase above the critical concentration.^29−33^ The cholesteric organized CNC suspension is extremely stable with the presence of polymers, micro-/nanoparticles, and surfactants and can be further solidified into photonic films upon slow solvent evaporation.^34−40^ Several studies have shown that CNCs could be used as a robust LC building block for designing custom-tailored functional materials in the dry state;^41−43^ in contrast, few works have focused on the CNC colloidal self-assembly as LC emulsions. Owing to the nanoparticle stabilizing effect at the liquid–liquid interface, the aqueous CNC cholesteric phase can be confined directly within the suspended droplet (water-in-oil),^44^ in the inverted continuous phase (oil-in-water),^45^ or both phases (water-in-water),^46^ generating a immiscible LC-fluid interface with hierarchically ordered structures. To the best of our knowledge, no attempt has yet been made to develop a bicontinuous structured LC emulsion with cholesteric ordered CNCs and an interpenetrating network. The combination of nanoscale colloidal self-assembly of CNC with microscale bicontinuous confined geometries can offer a scalable platform in the development of LC emulsions with complex long-range ordered structures.

Herein, we present an all-aqueous bicontinuous structured LC emulsion with hierarchical cholesteric self-assembly and intraphase trapping of nanoparticles. In specific, we describe the use of an aqueous binary polymer system composed of polyethylene glycol (PEG) and dextran as the starting materials, in which the dispersed CNC can keep the cholesteric ordering with the presence of immiscible polymers. When agitated by ultrasonication, binary mixtures of CNC–PEG and CNC–dextran can form into micrometer scaled all-aqueous emulsions. Low-mass-ratio of PEG/dextran tends to form tortuous interpenetrating fluid domains with a 3D liquid network; meanwhile, the high-mass-ratio of PEG/dextran tends to form aspherical droplets owing to the ultralow interfacial tension. The dispersed CNCs display unequal affinity for the binary PEG and dextran phase owing to the balance of osmotic potential and chemical potential difference between the two phases, displaying intraphase trapping of CNCs within the aqueous PEG component rather than the dextran counterpart. In addition, an excess amount of CNCs can spontaneously self-assemble into a cholesteric organization in the PEG phase, whereas the CNCs in the dextran phase maintain the isotropic state and result in percolating bicontinuous structured LC emulsion, showing particle segregation-induced structural arrest of CNCs in one polymer domain.

## 2 Experimental Section

### 2.1 Materials and Apparatus

Cellulose nanocrystals with high sulfur content (CNC-HS, Figure S1) were purchased from CelluloseLab (Canada) and kept at 4 °C to avoid drying and bacterial growth. The CNC concentration of this aqueous gel is about 10 wt %, and the crystallinity index is 75.6%. Dextran from *Leuconostoc mesenteroides* (*M*_w_ = 35,000–45,000), poly(ethylene glycol) (PEG, *M*_w_ = 20,000), fluorescein isothiocyanate-dextran (FITC-Dextran, *M*_w_ = 40,000), and calcofluor white (CFW) stain were purchased from Sigma-Aldrich (Vancouver, Canada). Dextran is composed of approximately 95% α-D-(1,6) linkages. The remaining α-(1,3) linkages account for the branching of dextran. *M*_w_ of PEG is tested according to Ph. Eur. All the chemicals were used as received without further purification. De-ionized water (18.2 MΩ·cm) was obtained from the Millipore-purified water system.

The microstructure of the prepared emulsion was observed through the Olympus BX53 optical microscope with a 10× or 20× air objective in fluorescent, phase contrast, and polarized modes. A drop of fresh emulsion sample was placed on a microscope slide and covered with a glass coverslip, which was quickly fixed by wax to avoid evaporation. The excitation/emission spectra for CFW-labeled CNC and FITC-dextran were 365/435 and 493/517 nm, respectively.

Confocal laser scanning microscopy was performed with a Leica TCS SP5 microscope with Leica Application Suite AF using PMT detectors and a HCX PL APO CS 40.0 × 1.25 OIL UV objective with 405 nm laser for CNC-CFW excitation and 488 nm laser for FITC-dextran excitation. A 488 nm argon laser was used as the light source combined with a 20× or 40× oil immersion objective in the absence of fluorescent labeling for polarized imaging.^47^ The optical birefringence was collected in the transmission mode using a polarized laser and a linear polarized filter oriented perpendicular to the polarization plane of the incident light. The focused laser scanned the sample at a fixed depth and created a horizontal optical slice. By repeating the scans at different depths, we obtained a stacking of each optical slice (z-stack), generating a polarized 3D pattern. The images were analyzed using the software of Leica Application Suite X (LAS X).

The surface tension measurement was conducted on a Theta Flex 300-Pulsating Drop Tensiometer (Biolin Scientific, Sweden) through the pendent drop method at room temperature. The interfacial tension of the dextran–PEG system (γ_12_) was estimated by the Good–Girifalco equation (),^48^ where γ_1_,γ_2_ represent surface tensions of dextran and PEG solutions, respectively, and φ is Good’s interaction parameter. As both phases in a dextran–PEG system are aqueous, the molecular interactions between phases and at their interface are either dipole–dipole (water–water) or hydrogen bond (dextran–water and PEG–water).^49,50^ We used the assumption of φ ≈ 1 when γ_12_ ≪ γ_1,2_ in our calculation, which fits well with the experimental data analysis from Makkonen–Kurkela.^51^

The rheological behavior of bicontinuous emulsions were measured with a rheometer (MCR 302, Anton Paar, Germany) using a cylinder (DG 26.7). The viscosity was monitored using increasing shear rates, from 0.01 to 100 s^–1^. All the measurements were performed at 25 °C.

For dynamic measurements, the linear viscoelastic range was determined by a cylinder (DG 26.7) using a strain sweep (0.1 to 100%) at a fixed frequency (10 rad·s^–1^). Thereafter, a constant strain (0.5%) was applied, within the linear region, and the viscoelasticity was determined over a frequency range between 0.1 and 100 rad·s^–1^. From the dynamic mechanical spectra, the storage (*G*′) and the loss (*G*″) moduli were determined as a function of frequency. All the measurements were performed at 25 °C.

### 2.2 Preparation of CNC–PEG and CNC–Dextran Suspensions

The concentrated CNC gel was first diluted to 300 mL by adding Milli-Q water to generate a homogeneous suspension with a concentration of 6.0 wt % and then sonicated for 2 min in an ice bath and sealed in a bottle. After equilibrium for 3 days, the resulting suspension separated into two phases with an upper isotropic and bottom cholesteric phase.

After that, the CNC–PEG and CNC–dextran suspensions were prepared by dissolving given amounts of polymers into the bottom cholesteric phase of the CNC suspension (CNC: 6 wt %, PEG: 10 wt %, dextran: 10 or 15 wt %). The obtained mixture was vigorously stirred overnight at room temperature and left to stand for at least 1 day for further usage.

### 2.3 Preparation of All-Aqueous Bicontinuous Liquid Crystal Emulsions

To investigate the liquid crystal nature of CNC in the bicontinuous emulsions, the all-aqueous bicontinuous emulsion was prepared by mixing the CNC–PEG and CNC–dextran components through a microtip sonicator (Sonifier 450, Branson Ultrasonics Co., USA) operated at an input power of 40% strength following alternating on–off cycles (3–2 s, respectively) for 90 s in an ice bath. The PEG-to-dextran weight ratios were varied from 10/90, 20/80 to 30/70 for the prepared emulsions.

In order to confirm the hierarchical structure of the bicontinuous emulsion, FITC-dextran and CFW were used as fluorescent labels, respectively. Briefly, a certain amount of normal dextran and FITC-dextran mixtures (w/w = 100/0.5) were dissolved into CNC suspension with final dextran concentration of 10 wt %. Then, the fluorescent CNC–dextran suspension was mixed with the CNC–PEG suspension following previous procedures. After emulsifying, CNC was dyed with CFW stain (CFW/emulsion = 1/100, w/w) and equilibrated at room temperature for further usage.

## 3 Results and Discussion

### 3.1 Binary Polymer Mixtures with Demixed Water–Water Interface

**Figure 1:**
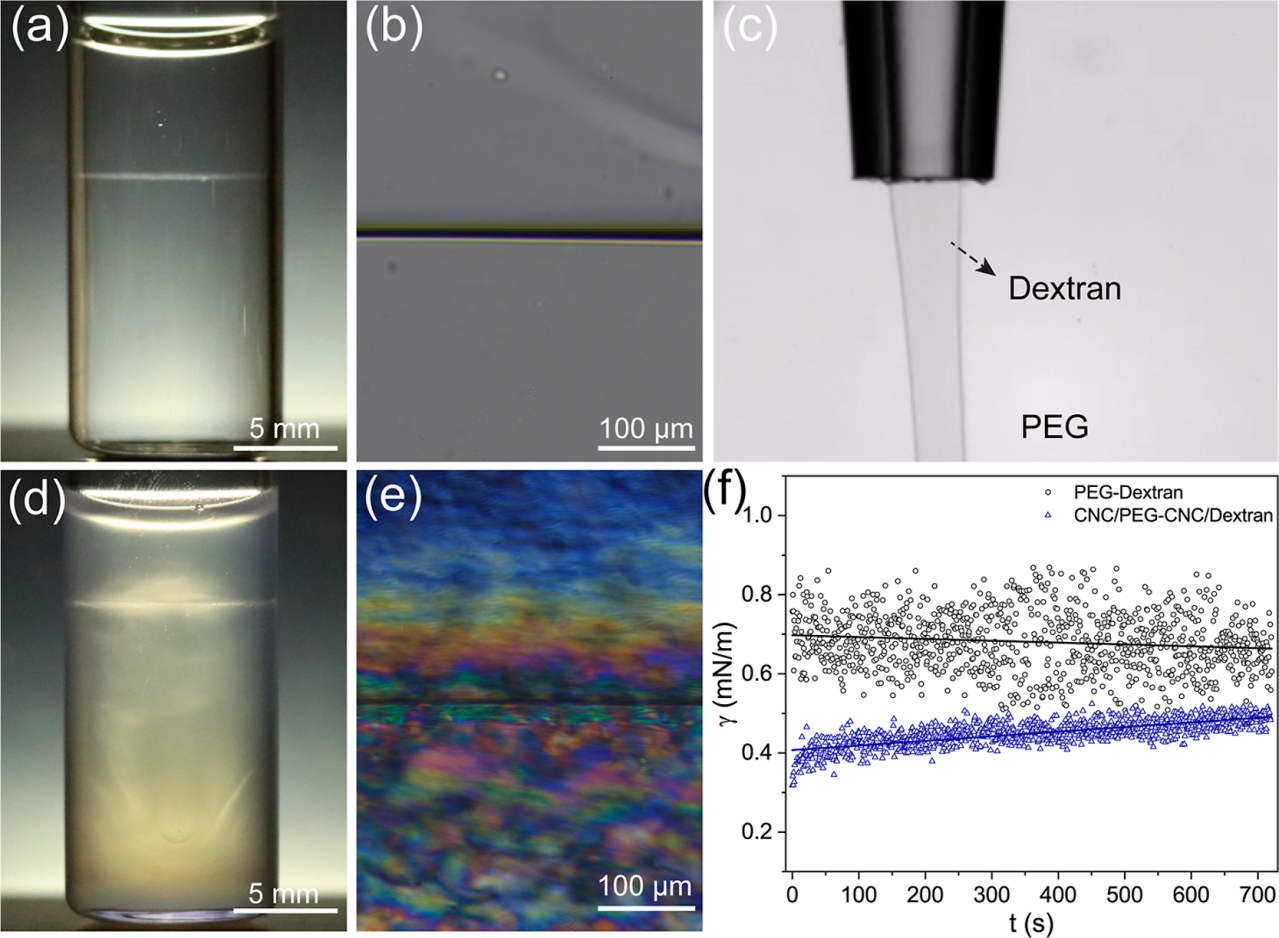
(a) Demixed PEG (upper) and dextran (bottom) binary solution with a sharp phase boundary. (b) High magnified microscopic optical image of the PEG/dextran interface. (c) Optical image of the liquid jet formation, rather than droplet due to the ultralow interfacial tension between PEG and dextran. (d) The corresponding cholesteric CNC–PEG (upper) and cholesteric CNC–dextran (bottom) binary suspension with LC–LC phase boundary. (e) High magnified polarized optical image of the phase boundary, showing anisotropic birefringent texture in both phases. (f) The measured interfacial tension of the all-aqueous PEG/dextran system over time with and without the addition of CNC.

The all-aqueous system is based on hydrosoluble water solutions of PEG (*M*_w_ = 20 kDa) and dextran (*M*_w_ = 45 kDa) at the concentration of 10 wt %, respectively, which are immiscible with each other (Figure 1a,b). The obtained PEG/dextran interface allows free diffusion and exchange of water, ions, and nanoparticles between the two aqueous phases,^52^ yielding a permeable interface that inspire the designing of a new kind of soft matter. In contrast to the oil–water system, the all-aqueous PEG/dextran system has an ultralow interfacial tension (γ) along the water–water interface, favoring the formation of liquid jets rather than droplets due to the Rayleigh–Plateau instability (Figure 1c and Movie S1).^53^ On the other hand, the CNC we used is obtained from sulfuric acid catalysis of wood-fiber paper pulp and displays a polydispersed rod-like morphology, whose length and width are around 80–180 and 5–10 nm, respectively (Figure S1). Adding nonadsorbing polymers of PEG and dextran as depletants into the CNC suspension can strongly affect the isotropic-cholesteric phase transition.^46^ Dispersion of CNC in the corresponding polymer phase (CNC: 6 wt %, PEG or dextran: 10 wt %) can produce a well-defined water–water interface, in which the CNC in each polymer phase self-assemble into a cholesteric LC ordering with the presence of immiscible polymers (Figure 1d,e). The measured helical pitch of cholesteric organized CNC in PEG and dextran phase corresponds to 7.3 and 6.5 μm (Figure S2), respectively, revealing the differences of the helical twisting force between neighboring CNC with the existence of polymer molecules.^54^ The ultralow interfacial tension of the PEG/dextran system can be estimated by the Good–Girifalco equation through measuring the individual surface tensions of PEG and dextran solutions. For the aqueous PEG/dextran interface, the measured interfacial tension is fluctuated around 0.70 mN/m, much lower than the regular oil–water interface. However, the interfacial tension of the PEG/dextran interface is further reduced to 0.45 mN/m in the presence of CNC, which can be ascribed to the inherent surface charge of CNC (ζ potential of −50 mV) that lead to interfacial charge and interfacial electrical potential difference across the water–water interface. Compared with the CNC-free polymer system, the interfacial tension is more stable with the addition of CNC, indicating the nanoparticle stabilizing effect at the liquid–liquid interface (Figure 1f). The homogeneous all-aqueous emulsion is produced by emulsifying the corresponding CNC–polymer LC aqueous mixtures (with the PEG/dextran mass ratio of 2:8) through ultrasonication (Figure S3). Immediately after emulsification, a small aliquot sample is transferred and sandwiched between two coverslips with the 0.12 × 9 mm adhesive spacer that seals the system and avoids water evaporation.

### 3.2 Morphological Characterization of Bicontinuous Structured LC Emulsion

**Figure 2:**
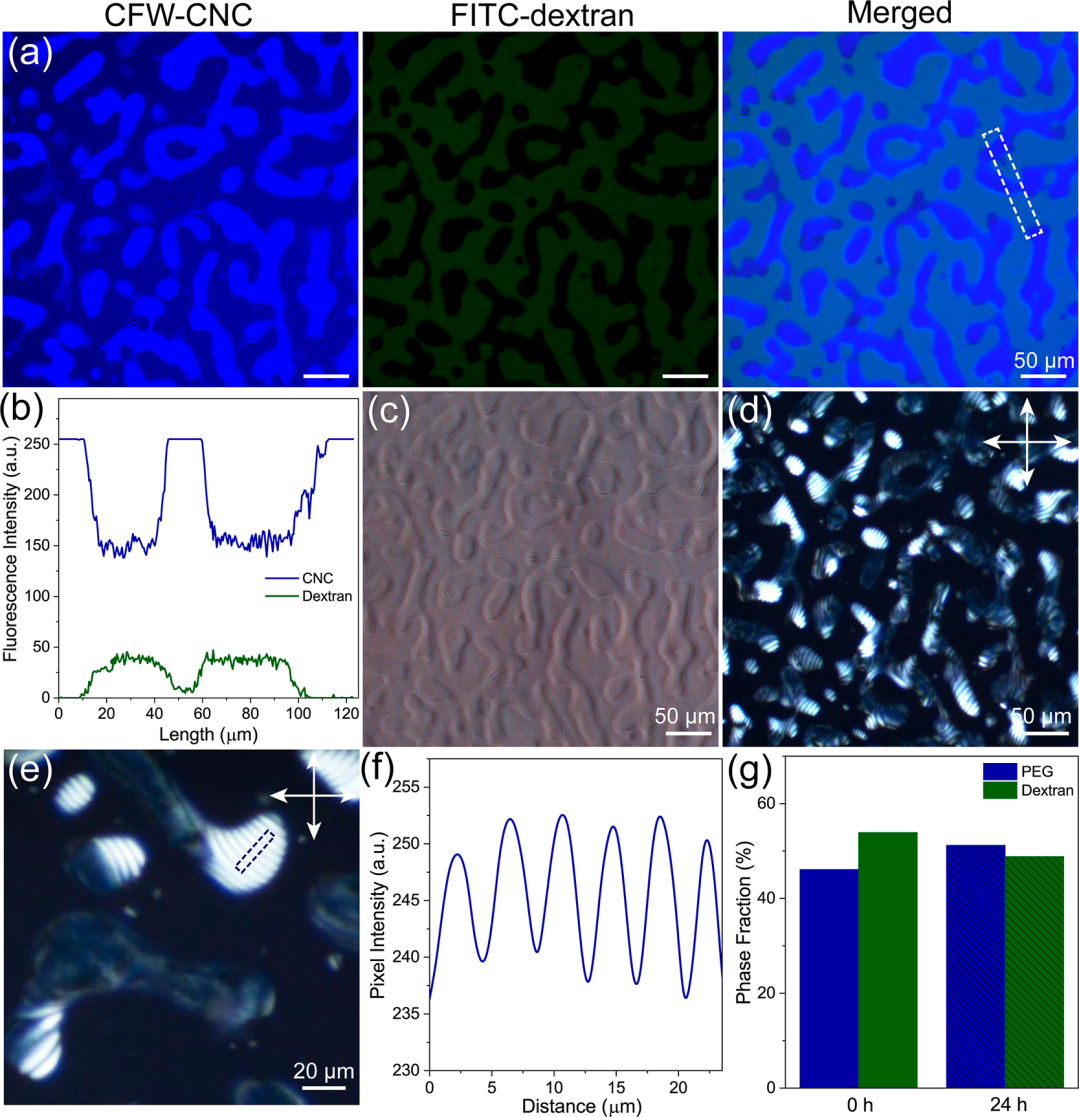
(a) Fluorescence microscopy images of the bicontinuous LC emulsion that focus on the CFW-labeled CNC, FITC-dextran, and their merged counterpart, respectively. (b) Fluorescent intensity analysis of the all-aqueous emulsion across the bicontinuous domain, demonstrating that the CNC nanoparticles are concentrated in the PEG region of the binary polymer mixture. (c,d) The bicontinuous structured LC emulsion under PCM and POM imaging modes, revealing that the PEG domain is in an anisotropic state, while the dextran domain remains isotropic. (e) Magnified POM image of the bicontinuous structured LC emulsion that highlights the fingerprint cholesteric texture in the PEG domain. (f) The helical pitch fluctuations in the dispersed PEG domain. (g) Calculated phase fractions of the PEG and dextran domains for bicontinuous emulsion before and after equilibrium, showing the high stability behavior of the sample.

Further insights into the colloidal self-assembly and internal structure of the prepared aqueous emulsion is given by analyzing their correspondent optical microscope characterization, providing a visual cue for the identification, organization, distribution, and stability of CNC nanoparticles in binary PEG/dextran polymer mixtures. To visualize the colloidal self-assembly in the emulsion structure, we have used CFW stain to modify the CNC surface and bind to 1-4 β-glycosidic bonds in glucose molecule,^55^ while fluorescein isothiocyanate (FITC) molecules are conjugated randomly to hydroxyl groups of dextran and yields FITC-dextran. The excitation and emission peaks of FITC-dextran are 490 and 520 nm, respectively, while for CFW, the excitation wavelength is 380 nm with the emission peak of 475 nm. Therefore, both CNC and dextran can be tagged with specific fluorophores, acting as individual fluorescent markers without significant cross influence. Figure 2a demonstrates a series of fluorescent microscopy images of the freshly prepared bicontinuous LC emulsion with the fluorescent marker ratio of 1:100. After homogenization, the obtained all-aqueous emulsion exhibits bicontinuous morphology with a large area of tortuous interpenetrating liquid domains on the order of several hundred micrometers (Figure S4). We first test the sample at the excitation wavelength of 365 nm because the fluorescence of CWF occurs best with ultraviolet light, whereas FITC-dextran remains inactivated. Surprisingly, the resulting bicontinuous emulsion displays nonuniform fluorescence appearance when the fluorescent markers are excited, namely, one of the tortuous domains is more fluorescent than the other (Figure 2a, left). When the excitation wavelength is switched to 493 nm, the fluorescent emission of FITC-dextran becomes dominant over CFW-labeled CNC. In such a condition, the corresponding fluorescence characterization clearly distinguishes the dextran domain from immiscible binary polymer mixture in which the PEG domain appears dark (Figure 2a, middle). It should be noted that a stronger relative fluorescence intensity tracks with a higher number density of CFW-labeled CNC and FITC-dextran. Therefore, we can conclude that the tortuous PEG domain holds more cellulose nanoparticles than the dextran counterpart, implying an uneven partitioning of CNC in the binary polymer mixture (Figure 2a, right). Quantitative CNC particle distribution characterization can be further evaluated from the fluorescent intensity across the multidomain structure of the bicontinuous emulsion. We calculate that the CNC concentrations in PEG and dextran domain are around 7.5 and 4.5 wt %, respectively. The obtained results demonstrate high CFW fluorescence intensity in the PEG domain and a deep concavity at the dextran domain; meanwhile, no fluorescence intensity fluctuation occurs for FITC-dextran in the same region (Figure 2b), which confirms the intraphase trapping of cellulose nanoparticles within the PEG domain in the bicontinuous emulsion. Previously, we showed that both PEG and dextran have the capacities to host CNC self-assembly in the aqueous emulsion, in which the suspended nanoparticles can be transferred through the PEG/dextran interface due to the imbalanced polymer contribution in osmotic pressure.^46^ In the current situation, the unequal affinity of CNC nanoparticles for PEG and dextran domain in bicontinuous emulsion is due to the balance of osmotic potential and chemical potential between the two polymer phases. Whereas the PEG domain has lower polymer potential than the dextran counterpart, and more CNCs are transferred into the PEG domain of the binary polymer mixtures and yield intraphase trapping of nanoparticles.

To push the analysis, the bicontinuous emulsion is further characterized with phase contrast microscopy (PCM) and polarized optical microscopy (POM), respectively, which allow us to determine how colloidal self-assembly of CNC occurs within the tortuous interpenetrating liquid domain. When the light waves pass through the all-aqueous bicontinuous emulsion, light–matter interactions within the PEG and dextran domains cause wave amplitude and phase change in a manner that depend on their corresponding refractive index, converting into image contrast differences to distinguish the binary polymer structures with similar transparency. Therefore, the PEG domain displays lower phase contrast than its dextran domain in bicontinuous emulsion owing to the CNC nanoparticle segregation (Figure 2c). In contrast, the POM image of the bicontinuous emulsion exhibits a birefringent long-range fingerprint texture within the tortuous PEG domain that is different from LC tactoid, while the dextran domain appears dark, revealing the cholesteric and isotropic organization of CNC nanoparticles in the two polymer domains (Figure 2d). High-magnified POM imaging shows that the obtained cholesteric CNC self-assembly is perfectly confined within the irregularly shaped bicontinuous PEG domain with a helical pitch of 8.5 μm (Figure 2e,f). Consequently, the above results indicate the hierarchical colloidal self-assembly of CNC in tortuous polymer domains and terms as bicontinuous structured LC emulsion with cholesteric-in-isotropic organization. Once segregation of nanoparticles occurs during the PEG/dextran phase separation, extra amount of CNC particles is constraint within PEG domains, further aggregated, and self-assembled into a fingerprint cholesteric organization above the critical concentration;^56^ by contrast, the CNC in the dextran domain remains isotropic due to its relatively low concentration. After sealing for 24 h, the as-prepared all-aqueous bicontinuous structured LC emulsion still preserves its tortuous morphology (Figures S5 and S6). The emulsion stability behavior is further evaluated by comparing the phase fraction ratio between dextran and PEG domains at different times, which almost keep constant at the ratio of 50:50 (Figure 2g), implying the stabilizing effect that due to the structural arrest of particles in one solvent domain.^13^

**Figure 3:**
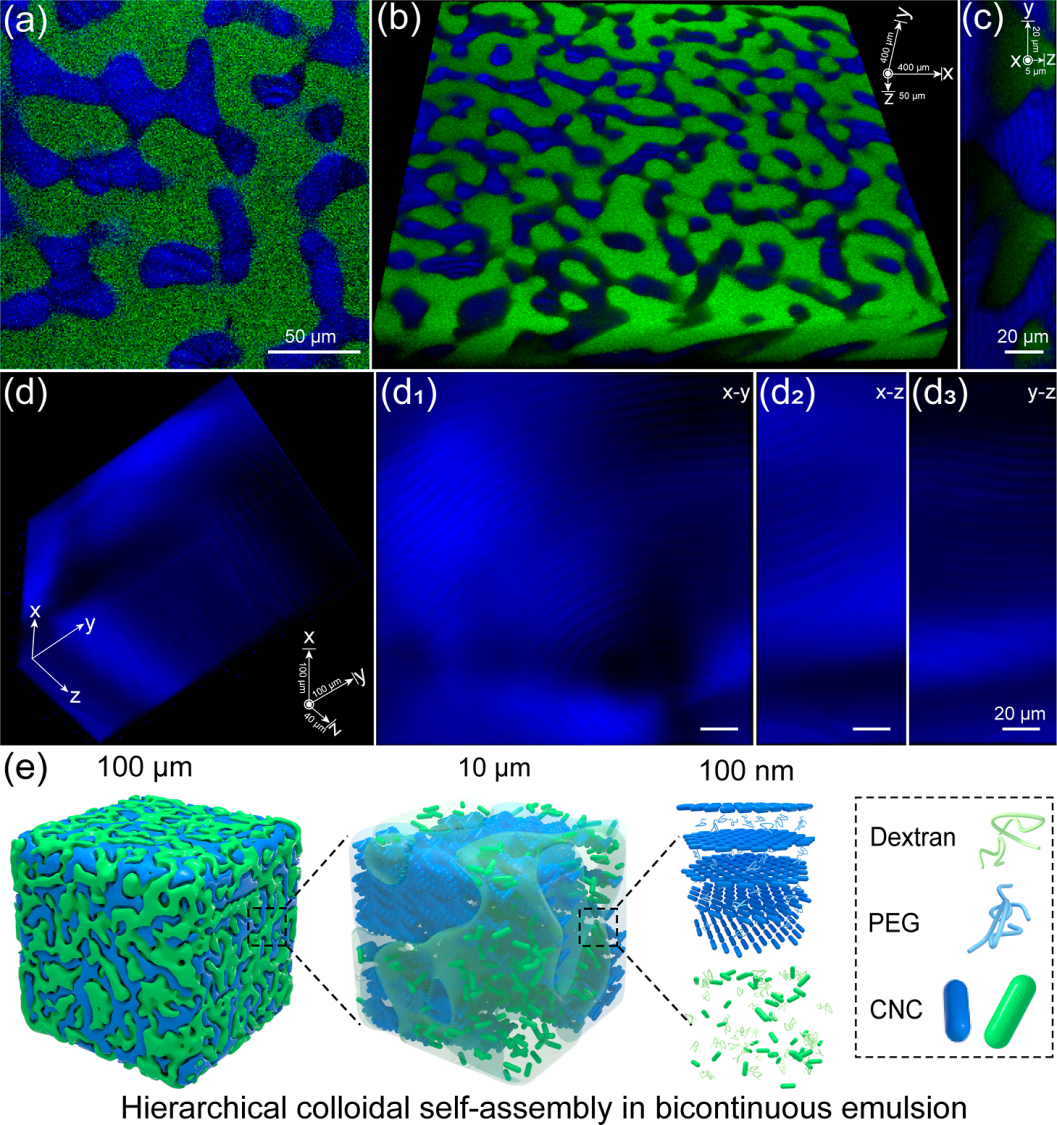
3D hierarchical structure of all-aqueous bicontinuous emulsion. (a) High-magnified LSCM image of the prepared bicontinuous emulsion that focus on a single plane with binary PEG and dextran domains, respectively. (b) The reconstructed confocal z-stack of the bicontinuous emulsion with 3D morphology. (c) Cross-section of the confocal z-stack of the bicontinuous emulsion taken normal to the focal plane, displaying the interconnected structures. (d) Polarized LSCM image of the reconstructed confocal z-stack of the bicontinuous emulsion that focus on the PEG domain, implying the fingerprint cholesteric structure. (d_1_–d_3_) Reconstructed focus planes from the LSCM image of the cholesteric PEG domain that demonstrate the 3D internal structure. (e) Illustration of the hierarchical colloidal self-assembly of CNC within multiscale structured bicontinuous emulsion with both cholesteric and isotropic domains, respectively.

With an understanding of the hierarchical CNC colloidal self-assembly in bicontinuous emulsion, we subsequently perform nonintrusive fluorescence and laser scanning confocal microscopy (LSCM) in the polarization mode at different depths across the sample to validate its 3D interconnected nature of the liquid network. The bicontinuous domains are clearly visible in a single plane of LSCM image (Figure 3a), displaying two tortuous interpenetrating liquid structures that correspond to the CFW-labeled CNC-rich PEG domain and FITC-dextran domain, respectively. The fluorescence z-stack of each scanning plane is overlapped and transformed into a 3D reconstruction of binary domains (Figure 3b and Movie S2), revealing the interconnected and bicontinuous nature of the obtained emulsion. Additionally, high-resolution LSCM fluorescence imaging with an orthogonal slice of this z-stack of the PEG domain further exhibits a microscale fingerprint texture within the tortuous interconnected liquid network (Figures 3c and S7 and Movie S3). Therefore, these results remind us to characterize the cholesteric CNC colloidal self-assembly through polarized LSCM imaging at different depths to reveal the hierarchical interconnected structure. Using the optical setup composed by polarized incident laser and a linear polarized filter with the perpendicular polarization plane, the optical birefringence of the bicontinuous LC emulsion can be imaged in the polarized confocal mode when light pass through the sample.^47^ The varying orientations of cholesteric colloidal self-assembly at different planes provides strong optical contrast to image the liquid crystalline CNC–PEG domain at different z-stacks, giving rise to 3D reconstruction of the cholesteric structure. High magnified polarized LSCM reconstruction of the PEG domain at varying z-stacks clearly exhibits a 3D fingerprint texture, confirming the hierarchical cholesteric self-assembly of CNC (Figure 3d). Specifically, the two-dimensional cross-sections (*x*–*y*, *y*–*z*, and *x*–*z* planes) of the PEG domain also reveals the varying orientations of the cholesteric domain with an interconnected structure, indicating the formation of a cholesteric LC network in the bicontinuous emulsion (Figure 3d_1−3_). The obtained optical results provide insights into the internal structure and colloidal self-assembly in the all-aqueous bicontinuous LC emulsion, revealing a 3D percolating cholesteric LC network in the isotropic phase due to the intraphase trapping and self-assembly of CNC within binary polymer domains.

Based on the above, Figure 3e is sketched to illustrate the multiscale hierarchical structure of the all-aqueous bicontinuous LC emulsion. CNC can be freely self-assembled into ordered structure in coexistence with the nonionic, hydrophilic polymers of PEG and dextran. The structural arrest of bicontinuous structures is due to the combination of the liquid–liquid demixing behavior of polymers and anisotropic repulsive interactions between rod-like CNCs. In which the binary polymer mixtures of PEG/dextran separate into two aqueous domains with a microscale 3D percolating interconnected liquid network that is commensurate with the observed bicontinuous structure.^57^ Driven by the balance of polymer potential between the two phases, the dispersed CNC is subjected to unequal affinity for the binary domains, in which the PEG domain overlaps with the CNC-rich region, while the dextran domain is depleted in CNC, resulting in intraphase trapping of CNC nanoparticles within the bicontinuous emulsion. Once at the critical concentration, the constraint CNC in the PEG domain can further nucleate and self-assemble into the cholesteric LC phase, while the CNC in the dextran domain remains isotropic, yielding bicontinuous structured LC emulsion with a characteristic length scale of several micrometers. In addition, the estimated separation distance between adjacent CNC nanoparticles in emulsion (∼50 nm) is much larger than the hydrodynamic radii of PEG and dextran (∼10 nm, Figure S8).^58^ Therefore, both two polymers can be entered between CNCs with nanoscopic helical and random co-assembly states, whereas the two polymer domains are thermodynamically incompatible to each other. The prepared cholesteric LC emulsion not only displays polymer-dependent bicontinuous morphology but also reveals nanoparticle segregation induced colloidal self-assembly, creating hierarchical ordered structures at varying range scales.

### 3.3 Emulsion Morphology Transition with Varying Polymer Ratios

**Figure 4:**
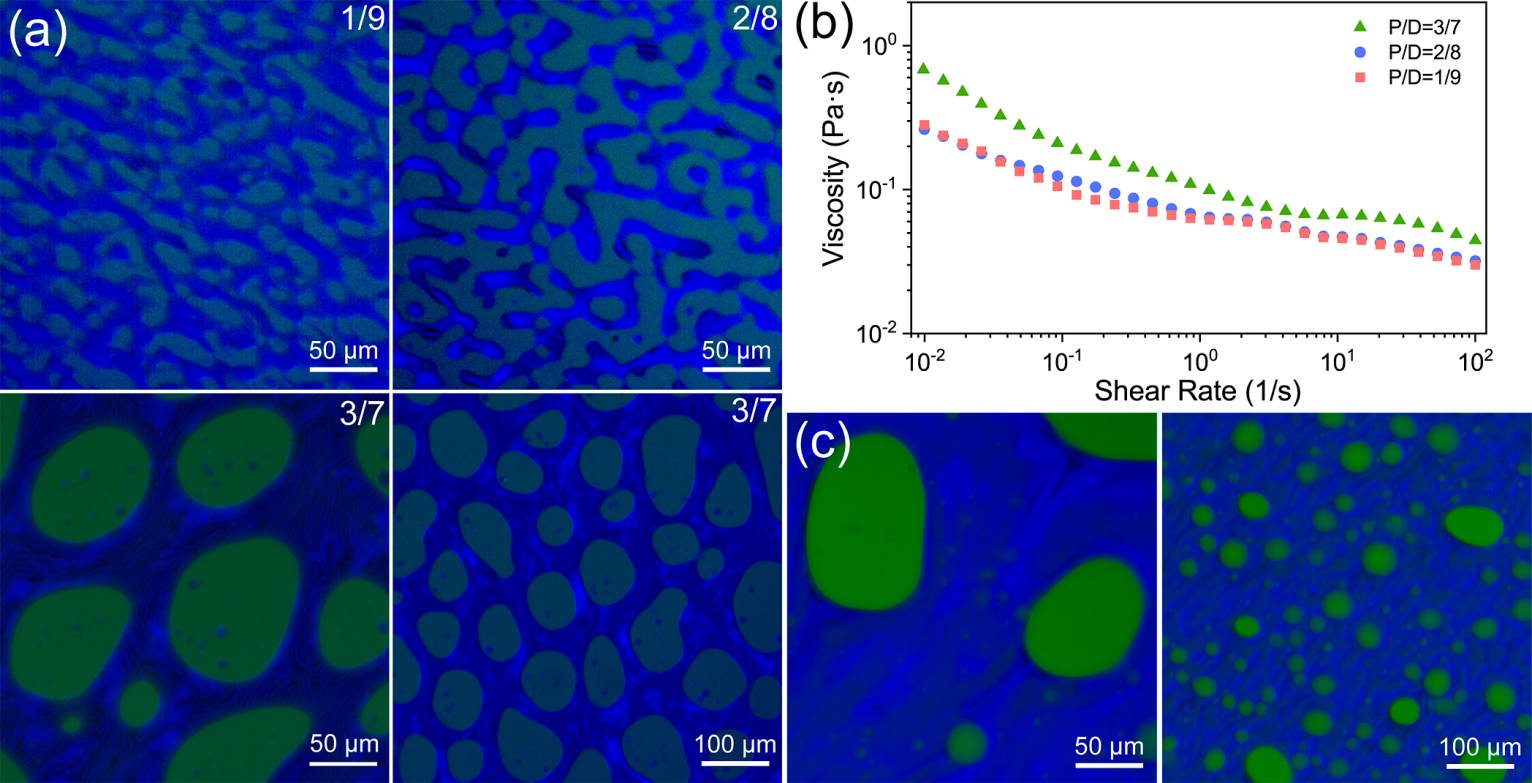
(a) Fluorescence images of the prepared emulsion with varying ratios of PEG/dextran, showing the morphology transition from bicontinuous to droplets. (b) Shear viscosity as a function of shear rate for the corresponding emulsions with varying morphologies. (c) Fluorescence images the all-aqueous emulsion with high content of dextran which display droplet morphology at different magnifications.

Apart from its bicontinuous structure, the emulsion morphology is strongly dependent on the polymer composition in aqueous mixtures. By adjusting the preparation protocols, it is possible to tune the emulsion morphology from bicontinuous to aspherical droplets by changing the mass ratio between PEG and dextran or the initial polymer concentration. To elucidate the emulsion morphology evolution, we first vary the initial mass ratio of PEG/dextran going from low to high (Figure S9). The prepared emulsions demonstrate tortuous bicontinuous domains at low-mass-ratio (1:9 and 2:8) and transform into droplet morphology when the mass-ratio is further increased (3:7), showing an aspherical FITC-dextran droplet in PEG continuous phase (Figure 4a). The viscosity of initial CNC–PEG and CNC–dextran suspension decreased with the increase of shear rates, indicating the shear-thinning behavior (Figure S10). After mixing, the corresponding rheological results of the obtained emulsions exhibit a typical non-Newtonian behavior. The variation of apparent viscosity as a function of shear rate is strongly dependent on the morphology of emulsion, even small changes in the microstructure of an emulsion lead to remarkable changes in the rheological behavior. For the emulsions with bicontinuous morphology, the relative viscosity decreases more slowly than its droplet counterpart at low shear rates and generates a plateau profile at high shear rates, which can be ascribed to the shear-thinning behavior and revealing the compression induced microstructural changes in different packing states of the liquid network (Figures 4b and S11). By contrast, increasing the dextran concentration to 15 wt % while fixed PEG/dextran mass-ratio at 2:8, the obtained emulsion remains in the droplet morphology (Figures 4c and S12), implying the polymer-dependent morphology transition between bicontinuous and droplet emulsion. These results can be explained by the liquid–liquid phase separation of an aqueous PEG/dextran mixture.^57^ When the polymer concentration is low, the aqueous mixture tends to form a single phase, whereas when the polymer concentration is high, the mixture demixed into two immiscible phases with the aqueous phase boundary. When the volume of one phase is much higher than the other, emulsion droplets are formed with either PEG-in-dextran or dextran-in-PEG assemblies. Whereas if PEG and dextran phase occupy similar volume fractions, a bicontinuous structured emulsion is formed with intraphase trapping and self-assembling of the suspending CNC nanoparticles into cholesteric organization within one phase to stabilize the liquid–liquid interface.

## 4 Conclusions

In summary, we have shown the formation of the all-aqueous bicontinuous structured LC emulsion through the combination of immiscible binary polymers and self-assembled CNC nanoparticles. Hierarchical colloidal self-assembly of CNC is achieved through intraphase trapping of nanoparticles within different polymer domains, displaying both 3D percolating cholesteric LC network in the PEG domain and isotropic interconnected CNC–dextran domain. Controllable emulsion morphology can be switched from bicontinuous to aspherical droplets by changing the mass ratio of polymers to control the liquid–liquid phase separation behavior. The obtained all-aqueous emulsion system providing a platform to design novel type of LC colloids with a tortuous bicontinuous morphology not only expands our knowledge to understand the interplay of colloidal self-assembly within multidomain structures but also paves the way for the designing of hierarchical ordered soft matter with diverse applications. We anticipate that the fundamental ideal presented in this study will be intriguing for other aqueous nanocolloid systems with different types of self-assembly and composition, bearing great potential to achieve more sophisticated structures than regular rod-like nanoparticles.

## Supporting Information Available

Experimental section; TEM image and XRD pattern of CNC; POM images of the binary PEG/dextran mixtures; photograph of the prepared all-aqueous emulsion; fluorescence and POM images of the freshly prepared and aged all-aqueous emulsion; 3D reconstructed z-stack of the LSCM image of the PEG domain; storage and the loss moduli of emulsions with varying PEG/dextran ratios; and apparent shear viscosity of PEG solution and dextran solution in the CNC suspension(PDF)
Drop dispersing dextran phase into continuous PEG phase (MP4)
Three-dimensional z-stack reconstruction of the all-aqueous bicontinuous emulsion with an interconnected structure (MP4)
Two-dimensional z-stack reconstruction of the all-aqueous bicontinuous emulsion with an interconnected structure at the y–z plane (MP4)
