# Supplementary material for: All-Aqueous Bicontinuous Structured Liquid Crystal Emulsion through Intraphase Trapping of Cellulose Nanoparticles
Source: Biomacromolecules. 2022 Dec 8;24(1):367–76. doi: 10.1021/acs.biomac.2c01177 (PMC9832472; doi:10.1021/acs.biomac.2c01177)
Supplement: Supplementary file 1 — bm2c01177_si_001.pdf [file bm2c01177_si_001.pdf]

Supporting Information

All-Aqueous Bicontinuous Structured Liquid Crystal Emulsion through Intraphase Trapping of Cellulose Nanoparticles

*Shasha Guo,[1, 2] Han Tao,[3] Guang Gao,[4] Sameer Mhatre,[2] Yi Lu,[2] Ayako Takagi,[2] Jun Li,[1] Lihuan Mo,[1] Orlando J. Rojas,[2, 3,] * and Guang Chu[3,] **

[1] School of Chemistry and Chemical Engineering, State Key Laboratory of Pulp and Paper Engineering, South China University of Technology, Guangzhou 510640, China

[2] Bioproducts Institute, Department of Chemical & Biological Engineering, Department of Chemistry and Department of Wood Science, The University of British Columbia, Vancouver, BC V6T 1Z3, Canada

[3] Bio-based Colloids and Materials, Department of Bioproducts and Biosystems, School of Chemical Engineering, Aalto University, Vuorimiehentie 1, 02510 Espoo, Finland

[4] Department of Cellular and Physiological Sciences, Life Sciences Institute, University of British Columbia, Vancouver, BC, V6T 1Z3, Canada

Corresponding Author:

Guang Chu, chuguang88@gmail.com, Tel: +358503080661

Orlando J. Rojas, orlando.rojas@aalto.fi, orlando.roja@ubc.ca, Tel: +1-604-822-3457

1. Experimental section

The morphology of CNC was conducted on FEI Tecnai G2S-Twin transmission electron microscopy (TEM) at an acceleration voltage of 80 kV. The prepared colloidal suspension (0.01 wt%, 5 μL) was deposited on the Cu200 formvar/carbon grid for 30 s and removed the extra amount of water with filter paper. Then the sample was stained with 2% uranyl acetate solution (5 μL) with the excess liquid removed by blotting with a filter paper and dried at room temperature.

X-ray diffraction analyses (XRD) of CNC was performed with a X-ray diffractometer (X'pert Powder, PANalytical, Holland) using a Cu Kα radiation at 40 kV and 40 mA. Scattered radiation was detected in the range of scattering angle (2θ) from 5° to 40° at a scan rate of 4°/min. The empirical crystallinity index (CrI) was calculated according to Eq. (1)[1]:

$$C_rI = \frac{I_{200} - I_{am}}{I_{200}} \times 100\% \qquad (1)$$

where, I_{200} is the maximum peak intensity at a 2θ angle (~22.5°), and I_{am} is the minimum diffraction at a 2θ angle of ~ 18°.

Hydrodynamic radii of PEG and Dextran aqueous suspension were measure using a Zetasizer Nano (ZS-90, Malvern Instruments, Worcestershire, U.K.).

2. Supporting figures

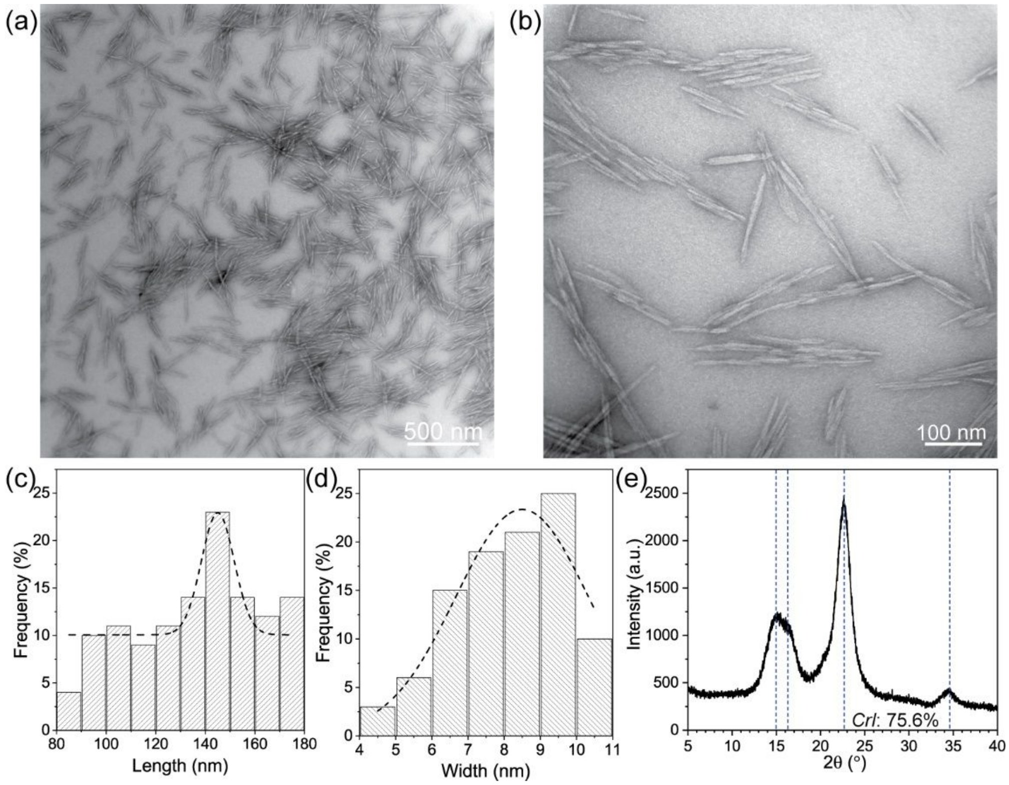

Figure S1. TEM image (a) (b) and histogram corresponding to the length (c) and width (d) of dilute CNC suspension. XRD pattern of CNC (e).

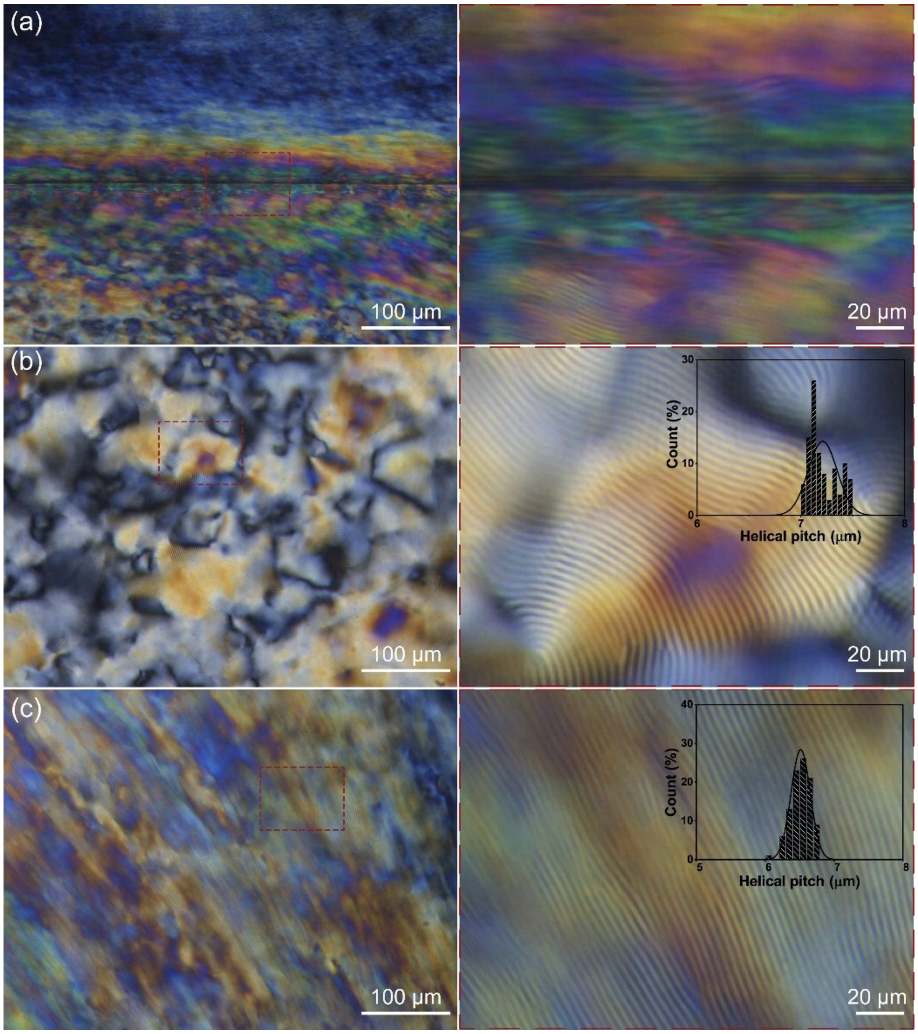

Figure S2. POM images of the binary PEG/dextran mixtures that focus on the interfacial region a), PEG phase b), and dextran phase c), displaying the cholesteric self-assembly of CNC at different magnifications.

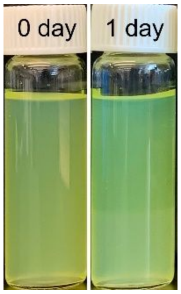

Figure S3. Photograph of the prepared all-aqueous emulsion before and after equilibrium with PEG/dextran ratio of 2:8.

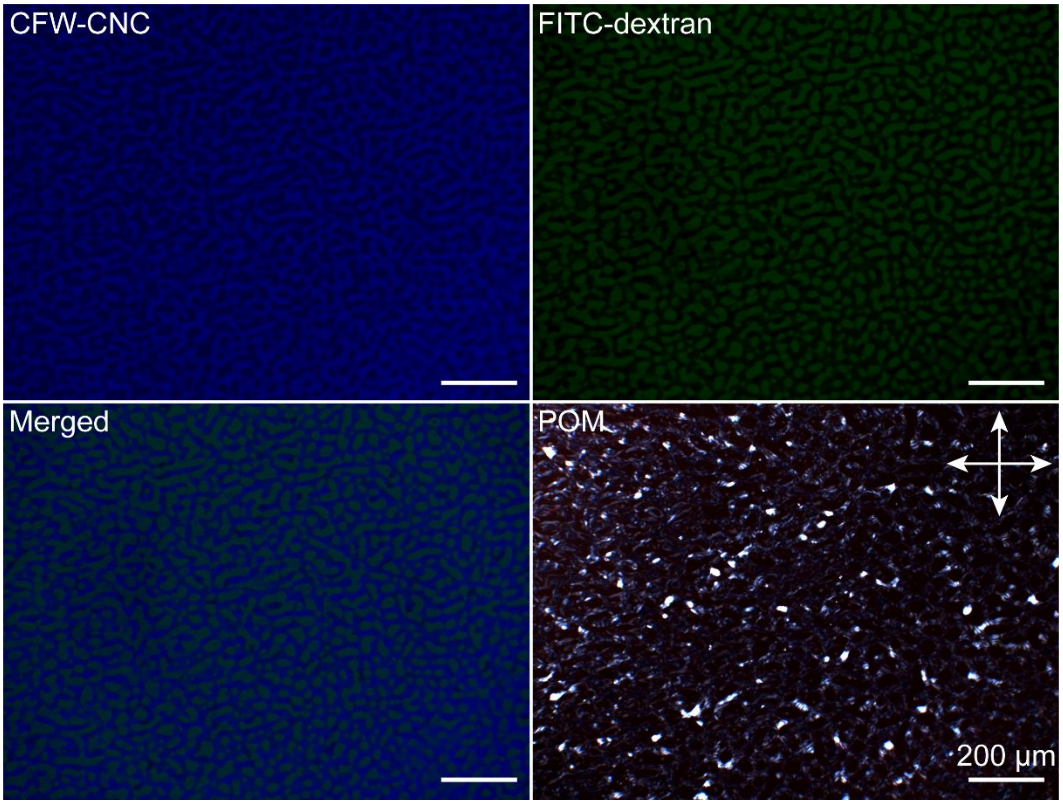

Figure S4. Fluorescence and POM images of the freshly prepared all-aqueous emulsion, showing tortuous bicontinuous morphology and anisotropic texture in the PEG domain.

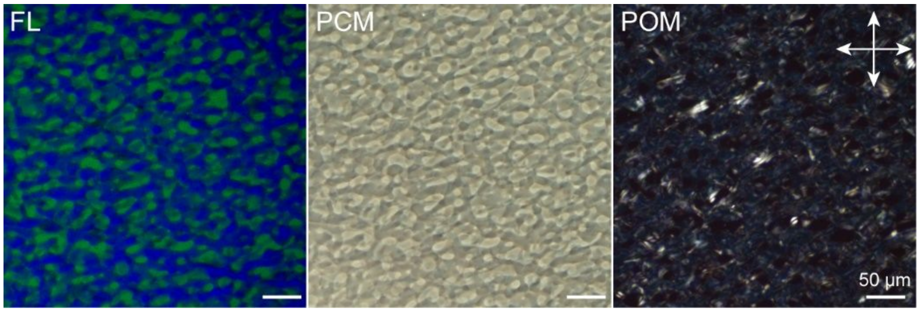

Figure S5. Fluorescence, PCM, and POM image of the aged all-aqueous bicontinuous emulsion that display anisotropy and interconnected structure.

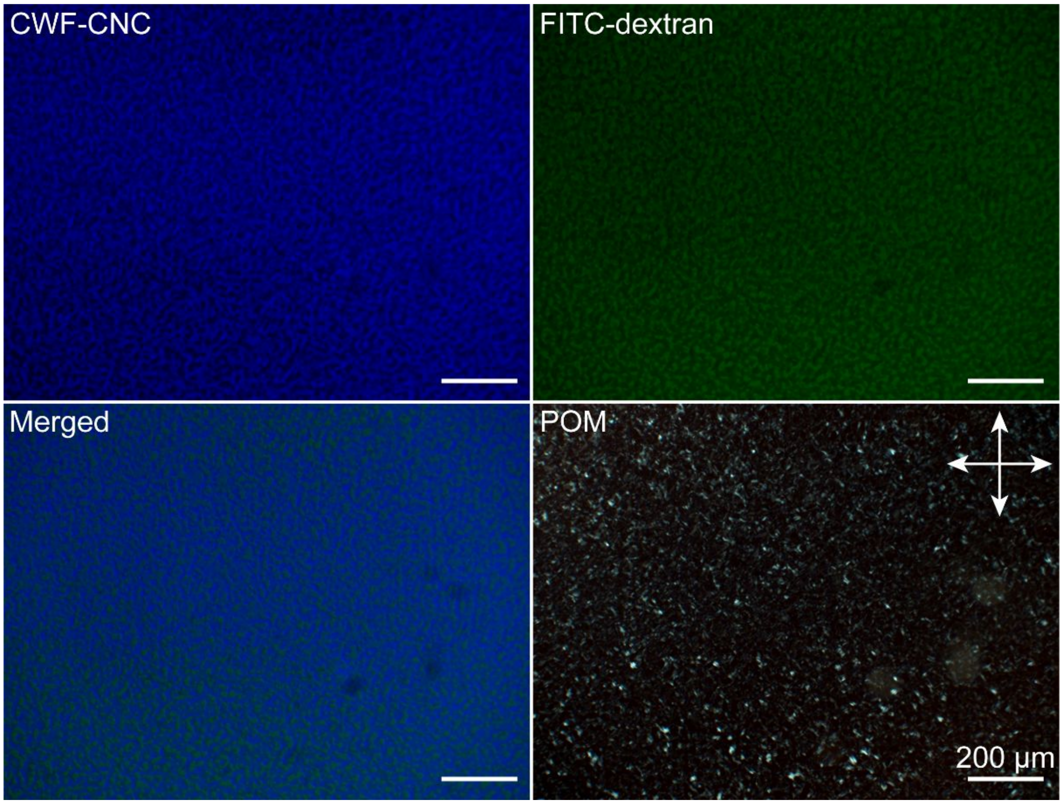

Figure S6. Low magnified fluorescence (which focus on the CFW-CNC, FITC-dextran, and their merged region) and POM images of the aged all-aqueous bicontinuous emulsion, demonstrating the high morphology stability.

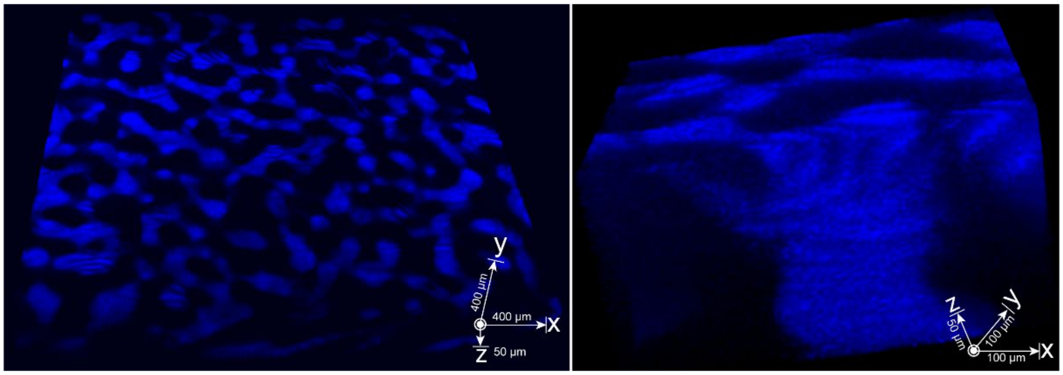

Figure S7. 3D reconstructed z-stack of the LSCM image of the PEG domain with different magnifications, showing the hierarchical cholesteric organization of CNC colloidal self-assembly.

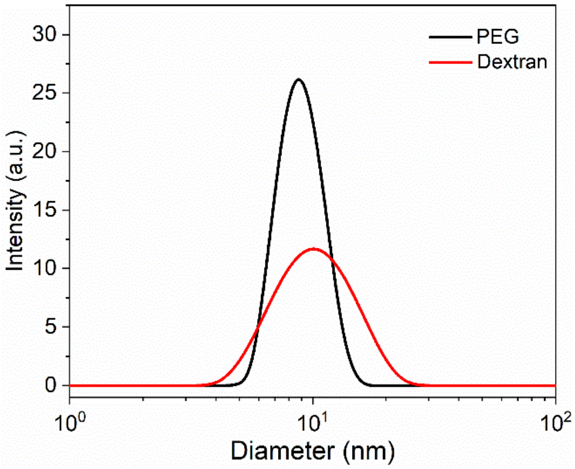

Figure S8. The hydrodynamic radii of PEG and dextran aqueous suspension based on dynamic light scattering measurement.

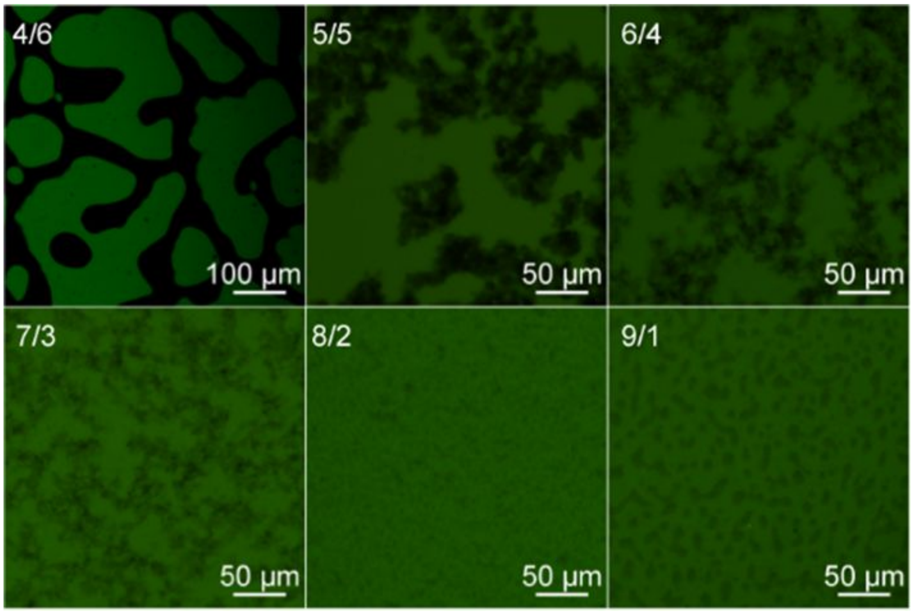

Figure S9. Fluorescence images of the prepared emulsions with varying ratios of PEG/dextran from 4/6 to 9/1.

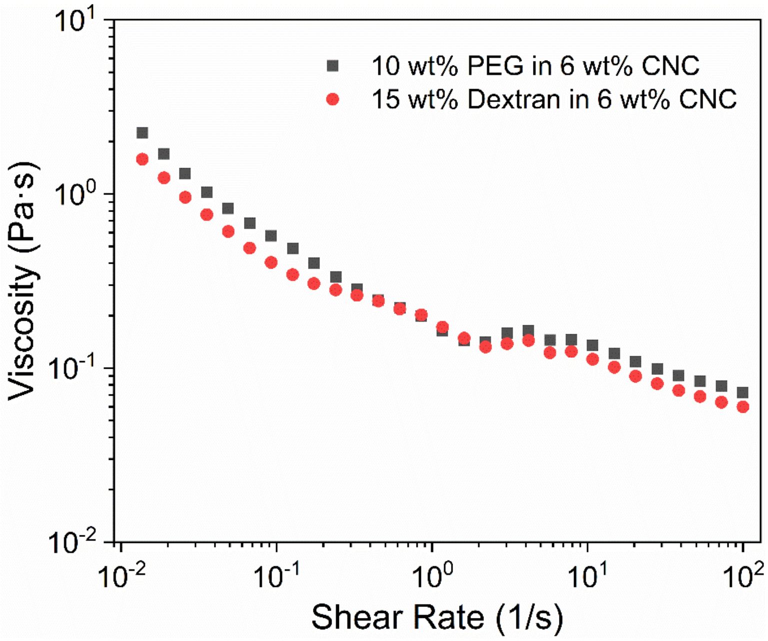

Figure S10. Apparent shear viscosity with shear rate for CNC-PEG and CNC-dextran suspensions at the CNC concentration of 6 wt% and polymer concentration of 10 wt%.

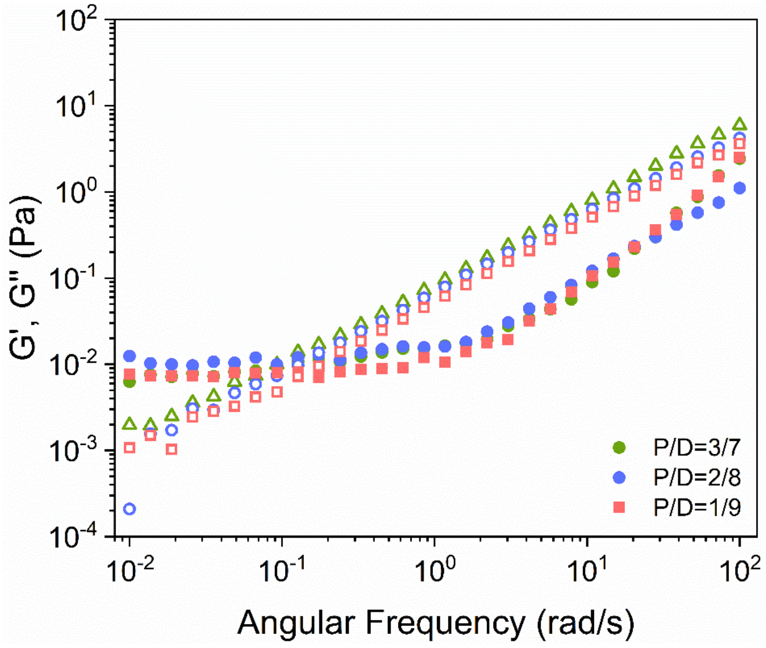

Figure S11. Evolution of the storage (G', filled symbols) and the loss (G'', open symbols) moduli of the corresponding emulsions with varying morphologies.

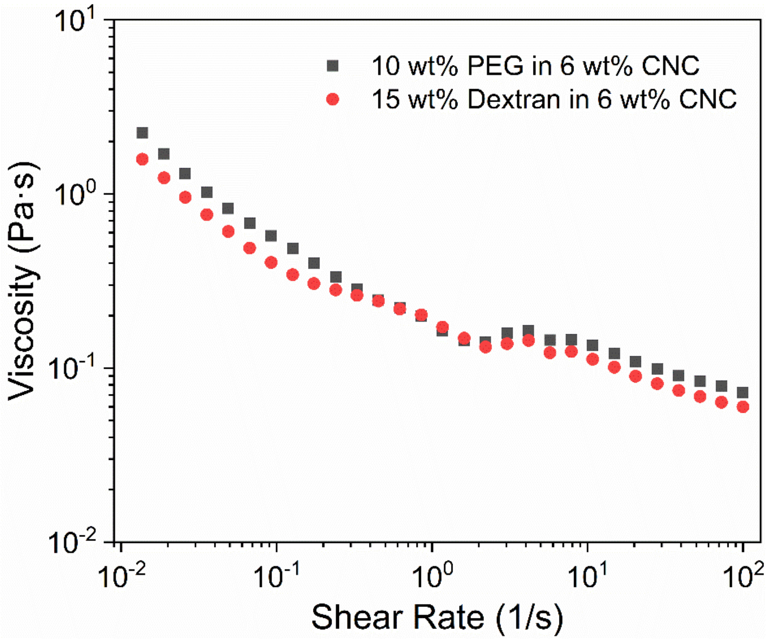

Figure S12. Apparent shear viscosity with shear rate for PEG solution and dextran solution in CNC suspension.

3. Supporting movies

Supplementary Movie S1: Drop dispersing dextran phase into continuous PEG phase, showing the formation of liquid jet with Rayleigh-Plateau instability that due to the ultralow interfacial tension between PEG and dextran. Videos are recorded and played at 30 fps.

Supplementary Movie S2: Three-dimensional z-stack reconstruction of the all-aqueous bicontinuous emulsion with interconnected structure.

Supplementary Movie S3: Two-dimensional z-stack reconstruction of the all-aqueous bicontinuous emulsion with interconnected structure at the y-z plane.

Reference

(1) Segal, L.; Creely, J. J.; Martin Jr, A.; Conrad, C. An empirical method for estimating the degree of crystallinity of native cellulose using the X-ray diffractometer. *Text. Res. J.* **1959,** 29, 786-794.
